# Five-year trend in secondary prevention medication prescription and risk factor control among patients with diabetes mellitus and cardiovascular diseases in Perak health clinics

**DOI:** 10.51866/oa.572

**Published:** 2024-07-31

**Authors:** Jazlan Jamaluddin, Mohd Azzahi Mohamed-Kamel, Nor Shazatul Salwana Din, Mohamad Zikri Mohamad-Isa

**Affiliations:** 1 MD, MMed (Fam Med), Klinik Kesihatan Selayang Baru, Jalan Sungai Tua, Batu Caves, Selangor, Malaysia. Email: jazlanjamaluddin@gmail.com; 2 MD, MMed (Fam Med), Klinik Kesihatan Lenggong, Jalan Besar, Kampung Batu Berdinding, Hulu Perak, Perak, Malaysia.; 3 MD, MMed (Fam Med), Klinik Kesihatan Kuala Selangor, Jalan Klinik, Kuala Selangor, Selangor, Malaysia.; 4 MBBS, MMed (Fam Med), Klinik Kesihatan Lintang, Sg. Siput (U), Lintang, Kuala Kangsar, Perak, Malaysia.

**Keywords:** Prescriptions, Cardiovascular diseases, Diabetes mellitus, Registries, Malaysia

## Abstract

**Introduction:**

Prescription of secondary prevention medications (SPMs) and effective control of cardiovascular risk factors (RFs) are crucial to reduce the risk of recurrent cardiovascular events, particularly in high-risk individuals including those with diabetes mellitus (DM). This study aimed to analyse the trends in SPM prescription and identify the factors associated with RF control among patients with DM and cardiovascular diseases in Perak health clinics.

**Methods:**

Data of patients with ischaemic heart disease (IHD) and cerebrovascular diseases (CeVDs) audited from 2018 to 2022, excluding those lost to follow-up, were extracted from the National Diabetes Registry. Descriptive and trend analyses were conducted. Multivariable logistic regression was utilised to identify the factors associated with RF control.

**Results:**

Most patients (76.7%) were aged ≥60 years and were Malays (62.3%). The majority had IHD (60.8%) and CeVDs (54.7%) for ≥5 years. SPM prescription increased significantly over the past 5 years. However, blood pressure (BP) and lipid control remained static. Good BP control was associated with a DM duration of ≥10 years and poor control with Malay ethnicity and prescription of two or three antihypertensives. Good DM control was associated with an age of ≥60 years and age at DM diagnosis of ≥60 years and poor control with Malay and Indian ethnicities, DM duration of ≥10 years and prescription of two or three and more glucose-lowering drugs. Poor lipid control was associated only with Malay and Indian ethnicities.

**Conclusion:**

SPM prescription has increased over time, but the achievement of treatment targets, particularly for lipid control, has remained poor and unchanged. Statin use is not associated with lipid control. The accessibility and availability of alternative lipid-lowering drugs must be improved to enhance overall RF control, especially lipid control, in patients with DM and cardiovascular diseases.

## Introduction

Diabetes mellitus (DM) is a chronic metabolic disorder with a significant impact on global health.^1^ It is associated with an increased risk of cardiovascular diseases (CVDs) and is a major contributor to morbidity and mortality worldwide. In Malaysia, the latest National Health and Morbidity Survey in 2019 showed that the overall prevalence of raised blood glucose levels among adults aged >18 years was 18.3%. Further, the prevalence of known DM was only 9.4%.^2^ Expectedly, mortality from CVDs remains high and continues to increase. Perak, a state in Malaysia, faces a considerable challenge in managing the burden of CVDs. According to the National Diabetes Registry (NDR) Report 2020, the proportion of patients in Perak diagnosed with ischaemic heart disease (IHD) increased within 1 year from 5.45% in 2019 to 5.46% in 2020.^3^ Similarly, the proportion of patients diagnosed with cerebrovascular diseases (CeVDs) increased within 1 year from 1.82% in 2019 to 1.90% in 2020. The prevalence of risk factors (RFs), such as hypertension, dyslipidaemia and DM, is high among the Perak population, contributing to the increasing incidence of CVDs. While primary prevention efforts are crucial, it is equally important to focus on secondary prevention to prevent recurrent events and complications among patients who have already experienced CVDs.

Secondary prevention strategies play a vital role in reducing the risk of recurrent cardiovascular (CV) events and improving patient outcomes.^4-6^ Prescription of and adherence to secondary prevention medications (SPMs) are crucial components of these strategies. Optimal SPMs for CVDs include antiplatelet agents, statins and angiotensin-converting enzyme inhibitors (ACEis) or angiotensin receptor blockers (ARBs). These medications have been proven to be effective in reducing CV events and improving long-term outcomes. However, the utilisation of SPMs can vary widely, and there may be disparities in the prescription patterns among different populations. International studies on community-level prescription of SPMs have shown conflicting results. In an audit undertaken in a primary care setting in the United Kingdom, 98.4% of patients with coronary artery disease received antiplatelet treatment in the community.^7^ Similarly, a study conducted in a university primary care clinic in Malaysia found that the prescription rate for SPMs ranged from 81.7% to 99.1 %.^8^ A Spanish study showed that 37.7%-85% of patients took SPMs.^9^ Larger community-based investigations have found a smaller proportion of SPMs being used in the studied communities.^10,11^ SPMs were administered to 20.4%-73.6% of patients with coronary artery disease in Europe and North America, with lower rates in the Middle East, South America and Asia.^10^ Chen et al. found that SPMs were used at substantially lower rates in China, ranging from 1.4% to 12.3%.^11^

Prescribing SPMs is crucial for reducing further morbidity and mortality. However, it is equally important to prioritise the effective management of other RFs, such as the low-density lipoprotein cholesterol (LDL-C) level, glucose level and blood pressure (BP). Current clinical practice often adheres to a ‘treat-to-target’ approach, where specific goals are established for these RFs, aiming for optimal control to reduce the risk of CV events. For example, stringent LDL-C targets have been set in guidelines to achieve maximal risk reduction.^12^ The ESC-EORP EUROASPIRE V registry findings indicate good prescription of and compliance to BP-, glucose- and lipid-lowering medications, including statins, among patients with coronary diseases in Europe.^13^ However, achieving control of important CV RFs, particularly the LDL-C level and BP, remains unmet in over half of patients. Similarly, the BARI 2D Trial, which included 49 clinical sites worldwide showed the same findings.^14^ Nonetheless, there remains a significant knowledge gap regarding the correlation between specific SPM prescription trends and the attainment of targeted control over key CV RFs. While the ‘treat-to-target’ strategy is widely endorsed, there is a paucity of research investigating the relationship between prescriptions of SPMs and their adequacy for the achievement of these key CV RF goals, especially in Malaysia. Therefore, this study aimed to investigate the trends in SPM prescription and RF control in Perak over 5 years and identify the factors associated with RF control. This study hopes to bridge the existing knowledge gap on the relationship between prescription practices and treatment goals, thereby improving secondary prevention strategies for enhanced patient care. By examining these trends and factors, healthcare providers, policymakers and researchers can gain valuable insights into the current practices and areas for improvement in secondary prevention strategies, especially regarding prescription habits. Understanding the relationship between SPM prescription and RF control is crucial for optimising patient care and reducing the burden of CVDs.

## Methods

This retrospective cross-sectional study was conducted over 5 years in Perak, which is one of the 13 states in Malaysia. All adults aged ≥18 years with IHD and CeVDs selected for audit in the NDR from 2018 to 2022 were included. The audit data were extracted from the NDR, a surveillance web-based registry database, to monitor DM control and clinical outcomes among patients receiving treatments from public healthcare facilities. The database has registry and clinical audit datasets. The registry dataset is continuously updated when new patients with DM are registered and when patients are lost to follow-up or death. Annually, a subset of patients with DM is randomly sampled for clinical audits. Clinical audits are conducted in August annually, whereby patients with type 2 DM from all public health clinics are randomly sampled. All active patients have an equal probability of being sampled regardless of whether they have been audited before. Patients with DM usually visit DM clinics several times each year, and their latest clinical information is used in the audits to represent their whole-year performance.

For this study, records of patients with IHD and/or CeVDs who were selected for audit each year in the NDR from 2018 to 2022 were extracted from the NDR database. Each patient’s record was screened for eligibility according to the inclusion and exclusion criteria. Patients who were lost to follow-up or had unavailable data when selected for audit were excluded. Since patients audited may differ each year, the data included in this study were limited to and measured at an interval of 1 year. Sociodemographic, clinical and medication prescription data were then used for analysis and interpretation. As for RF control, different guidelines suggest various ranges and cut-off points. Some patients maintain BP and LDL-C levels less than the lower limit of the RF target despite not receiving any related treatment. Additionally, patients may present with both IHD and CeVD together, rendering the establishment of highly specific treatment targets impractical. Since our sample had been diagnosed with DM, we followed the target recommended by the latest Clinical Practice Guidelines on the Management of Type 2 DM in Malaysia. According to these guidelines, the RF control targets for patients with CVDs are as follows: BP of <140/80 mmHg, glycosylated haemoglobin (HbAlc) level of ≤8.0% and LDL-C level of <1.4 mmol/L. Other clinical characteristics were categorised according to the recommendations of local guidelines.^12,15^

The sample size was calculated using OpenEpi, version 3.01, update 47 on 06/04/2013, for Sample Size for Frequency in a Population. Around 400 patients diagnosed with IHD and CeVDs underwent annual audits in the NDR over 5 years, resulting in a cumulative total of approximately 2000 patients. According to Baharudin et al., the percentage of patients prescribed antiplatelets, statins and ACEis/ARBs ranges from 81.7% to 99.1%.^8^ Therefore, approximately 14-207 patients are required to be studied to estimate the prevalence of SPM prescription among patients with DM, with an absolute precision of ±5% and a two-sided confidence interval (CI) of 95%. However, we included all adult patients with CVDs selected for audit in the NDR from 2018 to 2022 according to the inclusion and exclusion criteria, totalling around 2000 patients.

Data were analysed using IBM SPSS Statistics for Windows, version 27.0 (Armonk, NY: IBM Corp.). Data of patients with IHD and CeVDs in the NDR were de-identified and analysed as a cohort. Missing data were treated with listwise deletion in subsequent analyses. Data were summarised as numbers and percentages. Differences between groups were evaluated using the chi-square test or Fisher’s exact test, as appropriate. The Cochrane-Mantel-Haenszel test for trend was used to examine the temporal trends in RF control and SPM prescription from 2018 to 2022. A multivariable logistic regression analysis was performed with the target BP, HbA1c level and LDL-C level as the dependent variables considered separately to determine the factors associated with RF control among patients with DM and CVDs. Multiple logistic regression analyses using backward, forward and enter regression techniques were used, and variables with P-values of <0.25 were included in the final model. Only the most parsimonious model that determined the factors associated with good RF control for the overall sample was selected. Collinearity between variables was ruled out before covariates were introduced in the model. Goodness-of-fit was tested using the Hosmer-Lemeshow test, and odds ratios with 95% CIs were computed. All reported P-values were two-sided, and P-values of <0.05 were considered to indicate statistical significance.

## Results

In the past 5 years, 33,103 patients were audited, among whom 2307 fulfilled the inclusion criteria for this study. Of them, 1770 (76.7%) were >60 years old, while 1438 (62.3%) were of Malay ethnicity. Most patients had DM for ≥10 years (56.4%) and IHD (78.0%), while only 28.1% had CeVDs. The majority were prescribed antihypertensives (90.6%), glucose-lowering drugs (GLDs) (95.4%) and lipid-lowering drugs (LLDs) (87.6%). Among the three main RF control targets, the minority of the patients (10.6%) reached an LDL-C level of <1.4 mmol/L, whereas the majority achieved an HbA1c level of ≤8.0% (63.3%) and a BP of <140/80 mmHg (66.7%) (Table 1).

**Table 1:** Sociodemographic and clinical characteristics of the patients with DM and cardiovascular diseases.

Characteristics	n (%) or Median [IQR]
**Age, year**	
<60	537 (23.3)
>60	1770 (76.7)
**Sex**	
Female	1156 (50.1)
Male	1151 (49.9)
**Ethnicity**	
Malay	476 (20.6)
Chinese	1438 (62.3)
Indian	378 (16.4)
Others	15 (0.7)
**Duration of DM, year**	
<10	1005 (43.6)
≥10	1302 (56.4)
**Age at DM diagnosis, year**	
<60	1455 (63.1)
≥60	852 (36.9)
IHD	1780 (78.0)
**Duration of IHD, year**	
<5	697 (39.2)
≥5	1083 (60.8)
CeVD	632 (28.1)
**Duration of CeVD, year**	
<5	277 (45.3)
≥5	334 (54.7)
**Comorbidities**	
Erectile dysfunction	101 (22.5)
Retinopathy	492 (22.3)
Nephropathy	604 (26.9)
Diabetic foot ulcer	58 (2.6)
Amputation	18 (2.3)
Hypertension	2153 (93.6)
Dyslipidaemia	2083 (90.9)
Smoker	132 (9.5)
Waist circumference, cm	82 [16]
Normal	399 (28.4)
Elevated (Men: >90, Women: >80)	1004 (71.6)
Body mass index, kg/m^2^	26.6 [6.23]
<18.5 (Underweight)	269 (13.6)
18.5-22.9 (Normal)	32 (1.6)
23.0-27.4 (Overweight)	838 (42.4)
≥27.5 (Obese)	839 (42.4)
**Blood pressure, mmHg**	
<140/80	1488 (66.7)
≥140/80	743 (33.3)
Fasting blood glucose level, mmol/L	6.8 [3.1]
≤7.0	904 (55.3)
>7.0	731 (44.7)
HbA1c level, %	7.2 [2.6]
≤8.0	1405 (63.2)
>8.0	817 (36.8)
Total cholesterol level, mmol/L	4.5 [1.5]
≤5.2	1559 (73.2)
>5.2	571 (26.8)
Triglyceride level, mmol/L	1.4 [0.9]
≤1.7	1403 (66.6)
>1.7	703 (33.4)
HDL-C level, mmol/L	1.2 [0.4]
Normal	1188 (62.0)
Low (Men: <1.0, Women: <1.3)	728 (38.0)
LDL-C level, mmol/L	2.4 [1.3]
<1.4	203 (10.6)
≥1.4	1705 (89.4)
Creatinine level, mmol/L	87 [43]
eGFR category, mL/min/1.73 m^2^	74.3 [39.1]
G1 (≥90)	609 (28.8)
G2 (60-89)	829 (39.1)
G3 (30-59)	574 (27.1)
G4 (15-29)	81 (3.8)
G5 (<15)	25 (1.2)
**Proteinuria**	
Negative	1151 (66.5)
Positive	579 (33.5)
**Microalbuminuria**	
Negative	936 (70.3)
Positive	396 (29.7)
**Number of antihypertensives taken**	
0	218 (9.4)
1	628 (27.2)
2	761 (33.0)
3	492 (21.3)
≥4	25 (1.1)
**Number of GLDs taken**	
0	107 (4.6)
1	874 (37.9)
2	1179 (51.1)
≥3	147 (6.4)
**Number of LLDs taken**	
0	286 (12.4)
1	2007 (87.0)
2	14 (0.6)

IQR, interquartile range; DM, diabetes mellitus; IHD, ischaemic heart disease; CeVD, cerebrovascular disease; HbA1c, glycosylated haemoglobin; HDL-C, high-density lipoprotein cholesterol; LDL-C, low-density lipoprotein cholesterol; GLD, glucose-lowering drug; LLD, lipid-lowering drug.

**Figure 1:**
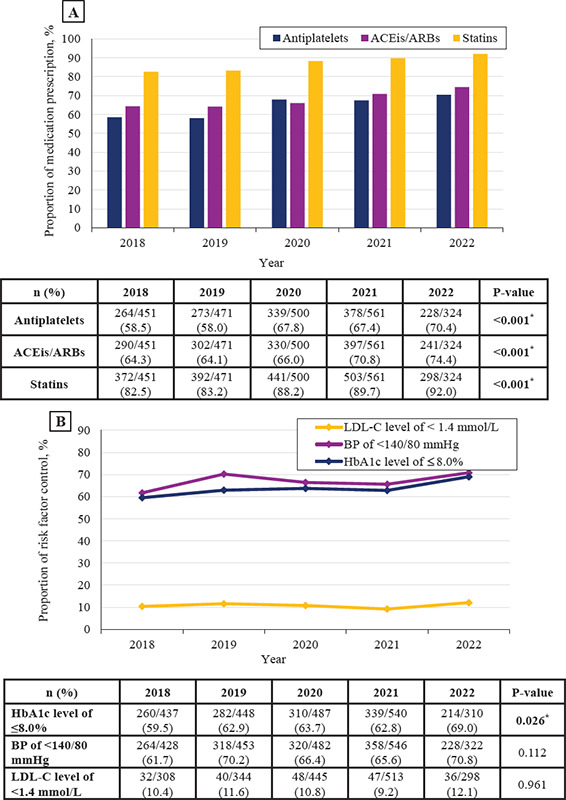
Trends in the prescription of secondary prevention medications (A) and control of risk factors (B) over 5 years. *Statistical significance at P<0.05; ACEi, angiotensin-converting enzyme inhibitor; ARB, angiotensin receptor blocker; BP, blood pressure; HbAlc, glycosylated haemoglobin; LDL-C, low-density lipoprotein cholesterol.

In the past 5 years, there was a significant increase in the prescription of SPMs, including antiplatelets (P<0.001), statins (P<0.001) and ACEis/ARBs (P<0.001). However, the RF control targets including a BP of <140/80 mmHg (P=0.112) and LDL-C level of <1.4 mmol/L (P=0.961) remained unchanged, while the trend in the target of an HbA1c level of ≤8.0% significantly increased (P=0.026). The trend in LDL-C control remained the lowest within 5 years (Figure 1).

In the multivariable analysis, good BP control was associated with an age of ≥60 years and a DM duration of ≥10 years, while poor BP control was associated with male sex, obesity and prescription of statins (Table 2). Good DM control was related to an age of ≥60 years and age at DM diagnosis of ≥60 years, while poor DM control was related to Malay and Indian ethnicities, DM duration of ≥10 years and prescription of two or three GLDs (Table 3). Conversely, poor LDL-C control was associated with only Malay and Indian ethnicities (Table 4).

**Table 2:** Factors associated with good blood pressure control among the patients with DM and cardiovascular diseases.

Characteristics	OR (95% CI)	P-value	aOR (95% CI)	P-value
**Age, year** <60 ≥60	Reference 2.02 (1.65, 2.47)	**<0.001** ^*^	Reference 1.60 (1.22, 2.10)	**<0.001** ^*^
**Sex** Female Male	Reference 0.75 (0.63, 0.90)	**0.002** ^*^	Reference 0.70 (0.55, 0.89)	**0.003** ^*^
**Ethnicity** Malay Chinese Indian Others	Reference 0.69 (0.55, 0.87) 0.87 (0.65, 1.17) 0.51 (0.17, 1.51)	0.001^*^ 0.363 0.224		
**Duration of DM, year** <10 ≥10	Reference 1.58 (1.33, 1.89)	**<0.001** ^*^	Reference 1.47 (1.16, 1.86)	**0.002** ^*^
**Age at DM diagnosis, year** No Yes	Reference 1.21 (0.98, 1.49)	0.072		
**Cerebrovascular disease** No Yes	Reference 0.82 (0.68, 1.00)	0.050		
**Number of comorbidities** 1 2 >3	Reference 1.37 (0.59, 3.18) 1.17 (0.53, 2.56)	0.465 0.703		
**Waist circumference** Normal Elevated	Reference 0.80 (0.62, 1.02)	0.074		
**Body mass index** Underweight Normal Overweight Obese	Reference 0.63 (0.29, 1.38) 0.79 (0.57, 1.08) 0.51 (0.37, 0.70)	0.251 0.136 <0.001^*^	Reference 0.67 (0.23, 1.91) 0.86 (0.58, 1.27) 0.56 (0.38, 0.82)	0.449 0.439 0.003^*^
**Number of antihypertensives taken** 0 1 2 3 >4	Reference 0.81 (0.56, 1.18) 0.79 (0.55, 1.13) 1.03 (0.70, 1.51) 0.97 (0.62, 1.50)	0.271 0.197 0.875 0.882		
**Prescription of antiplatelets** No Yes	Reference 1.00 (0.83, 1.20)	0.967		
**Prescription of statins** No Yes	Reference 0.68 (0.51, 0.92)	**0.012** ^*^	0.64 (0.43, 0.95)	**0.027** ^*^
**Prescription of ACEis/ARBs** No Yes	Reference 0.82 (0.67, 0.99)	**0.040** ^*^		6.8 [3.1] 904 (55.3) 731 (44.7)

* The model reasonably fitted well (Hosmer-Lemeshow test: P=0.901). Model assumptions were met. No significant interactions and multicollinearity problems were noted. The model explained 4.7% (Cox and Snell R^2^) to 6.5% (Nagelkerke R^2^) of the variance in good blood pressure control. *Statistical significance at P<0.05; DM, diabetes mellitus; OR, odds ratio; aOR, adjusted odds ratio; CI, confidence interval; ACEi, angiotensinconverting enzyme inhibitor; ARB, angiotensin receptor blocker.

**Table 3:** Factors associated with good DM control among the patients with DM and cardiovascular diseases.

Characteristics	OR (95% CI)	P-value	aOR (95% CI)	P-value
**Age, year** <60 ≥60	Reference 2.13 (1.74, 2.60)	**<0.001** ^*^	Reference 1.67 (1.22, 2.28)	**0.001** ^*^
**Sex** Female Male	Reference 1.17 (0.98, 1.39)	0.070		
**Ethnicity** Malay Chinese Indian Others	Reference 0.51 (0.40, 0.64) 0.40 (0.30, 0.54) 2.10 (0.47, 9.44)	<0.001^*^ <0.001^*^ 0.334	Reference 0.53 (0.38, 0.73) 0.51 (0.34, 0.75) 1.31 (0.24, 7.27)	<0.001^*^ <0.001^*^ 0.756
**Hypertension** No Yes	Reference 1.66 (1.18, 2.34)	**0.004** ^*^		
**Dyslipidaemia** No Yes	Reference 0.93 (0.69, 1.27)	0.660		
**Duration of DM, year** <10 ≥10	Reference 0.51 (0.42, 0.61)	**<0.001** ^*^	Reference 0.54 (0.41, 0.70)	**<0.001** ^*^
**Age at DM diagnosis, year** <60 ≥60	Reference 2.96 (2.43, 3.60)	**<0.001** ^*^	Reference 2.06 (1.51, 2.81)	**<0.001** ^*^
**Ischaemic heart disease** No Yes	Reference 0.92 (0.74, 1.13)	0.422		
**Cerebrovascular disease** No Yes	Reference 1.21 (1.00, 1.47)	0.055		
**Waist circumference** Normal Elevated	Reference 0.75 (0.58, 0.96)	**0.021** ^*^		
**Body mass index** Underweight Normal Overweight Obese	Reference 1.62 (0.67, 3.93) 0.98 (0.73, 1.32) 0.69 (0.52, 0.92)	0.282 0.910 0.013^*^		
**Number of GLDs taken** 0 1 2 ≥3	Reference 1.91 (1.23, 2.95) 0.77 (0.50, 1.17) 0.36 (0.21, 0.61)	0.004^*^ 0.214 <0.001^*^	Reference 0.70 (0.23, 2.13) 0.33 (0.11, 0.99) 0.15 (0.05, 0.49)	0.534 0.049^*^ 0.002^*^
**Prescription of antiplatelets** No Yes	Reference 1.18 (0.98, 1.41)	0.078		
**Prescription of statins** No Yes	Reference 1.11 (0.86, 1.44)	0.414		
**Prescription of ACEis/ARBs** No Yes	Reference 1.02 (0.85, 1.23)	**<0.001** ^*^		

* The model reasonably fitted well (Hosmer-Lemeshow test: P=0.213). Model assumptions were met. No significant interactions and multicollinearity problems were noted. The model explained 1.4% (Cox and Snell R^2^) to 1.9% (Nagelkerke R^2^) of the variance in good DM control. *Statistical significance at P<0.05; DM, diabetes mellitus; OR, odds ratio; aOR, adjusted odds ratio; CI, confidence interval; GLD, glucose-lowering drug; ACEi, angiotensin-converting enzyme inhibitor; ARB, angiotensin receptor blocker.

**Table 4:** Factors associated with good low-density lipoprotein cholesterol control among the patients with DM and cardiovascular diseases.

Characteristics	OR (95% CI)	P-value	aOR (95% CI)	P-value
**Age, year** <60 ≥60	Reference 0.97 (0.69, 1.36)	0.844		
**Sex** Female Male	Reference 1.33 (0.99, 1.78)	0.060		
**Ethnicity** Malay Chinese Indian Others	Reference 0.62 (0.44, 0.87) 0.50 (0.31, 0.82) 1.55 (0.42, 5.73)	0.005^*^ 0.006^*^ 0.510	Reference 0.62 (0.44, 0.87) 0.50 (0.31, 0.82) 1.55 (0.42, 5.73)	0.006^*^ 0.008^*^ 0.510
**Hypertension** No Yes	Reference 1.96 (0.90, 4.26)	0.091		
**Dyslipidaemia** No Yes	Reference 1.06 (0.62, 1.81)	0.846		
**Duration of DM, year** <10 ≥10	Reference 1.05 (0.78, 1.41)	0.759		
**Age at DM diagnosis, year** <60 ≥60	Reference 0.98 (0.72, 1.32)	0.884		
**Ischaemic heart disease** No Yes	Reference 0.96 (0.67, 1.35)	0.794		
**Cerebrovascular disease** No Yes	Reference 1.00 (0.72, 1.38)	0.986		
**Waist circumference** Normal Elevated	Reference 1.04 (0.69, 1.58)	0.839		
**Body mass index** Underweight Normal Overweight Obese	Reference 0.59 (0.13, 2.62) 1.08 (0.67, 1.73) 0.79 (0.48, 1.28)	0.485 0.763 0.330		
**Number of LLDs taken** 0 1 2	Reference 0.53 (0.87, 0.56) Not available	0.534 0.999		
**Prescription of antiplatelets** No Yes	Reference 0.73 (0.54, 0.98)	0.726		
**Prescription of statins** No Yes	Reference 0.77 (0.51, 1.17)	0.224		
**Prescription of ACEis/ARBs No Yes**	Reference 1.15 (0.83, 1.58)	0.401		

* The model reasonably fitted well (Hosmer–Lemeshow test: P=1.000). Model assumptions were met. No significant interactions and multicollinearity problems were noted. The model explained 0.6% (Cox and Snell R^2^) to 1.2% (Nagelkerke R^2^) of the variance in good low-density lipoprotein cholesterol control. *Statistical significance at P<0.05; DM, diabetes mellitus; OR, odds ratio; aOR, adjusted odds ratio; CI, confidence interval; LLD, lipid-lowering drug; ACEi, angiotensin-converting enzyme inhibitor; ARB, angiotensin receptor blocker.

## Discussion

This study demonstrated good prescription of SPMs including antiplatelets, statins and ACEis/ARBs among patients with CVDs within the past 5 years. The prescription pattern significantly increased over the past 5 years, especially for antiplatelets and statins (P<0.001). This finding is comparable to that of a local study conducted at the primary care level, where 99.1% of patients with coronary artery disease received statins,^8^ and to that of a study performed at the secondary care level, where 98.2% of patients were already started on statin treatment upon discharge.^16^ The finding is in contrast with that of a study where among individuals who already had CVDs, 74.2% were not taking statins as a secondary prevention treatment, and 89.5% of those who had a high Framingham risk score were not prescribed statins as primary prevention treatment.^17^ However, the study was conducted on a larger sample size. Overall, most patients in this study achieved the BP (66.7%) and DM control targets (63.2%) over 5 years. This is reassuring since robust evidence from international and local guidelines has emphasised optimum BP, glycaemic and lipid control as an important secondary prevention strategy among patients with CVDs and DM.^15^ The reported proportions are higher than those in another local study in primary care, where about one-third of participants with a high CV risk met their targets for systolic BP (35.1%) and diastolic BP (46.7%).^18^ Other studies in Asia and Europe on the achievement of the BP target have shown various results, ranging from 5.5% to 70%.^19,20^ The proportion of patients achieving the treatment goal for DM is larger in this study than in another study conducted in Malaysia.^17^ This difference can likely be attributed to the stricter treatment target used. Since the study population had a median HbA1c level of 7.0%, which is lower than that in our study, most patients would have reached the DM treatment goal if the target had been increased to 8.0%.

In this study, the LDL-C treatment target was not achieved in most patients (89.4%), with no significant changes over 5 years. The latest Malaysian guidelines align with the 2019 European Society of Cardiology and European Atherosclerosis Society recommendations, setting LDL-C goals of 1.4 and 1.8 mmol/L for very high- and high-risk individuals, respectively, and <2.6 mmol/L for moderate-risk individuals.^12,15^ Despite nearly 92% of patients being prescribed statins, only a small fraction achieved this stringent target. In contrast, Baharudin et al. reported that 60.2% of high CV-risk patients met the less stringent LDL-C target of <2.6 mmol/L based on the treatment target in the older Malaysian guidelines.^18^ Similarly, Vrablik et al. found that only 20.5% of patients, including 19.4% of very high-risk patients and 28.1% of high-risk patients, met the 2019 European guidelines’ LDL-C control target.^21^ Among very high-risk patients on statin monotherapy, only 5.4% on medium-intensity statins and 11.4% on high-intensity statins reached their LDL-C goals. The study highlighted a significant issue: Despite good treatment adherence, 61.5% of substantially high-risk patients who did not achieve the LDL-C treatment targets had physicians who did not consider changing the treatment regimen. This contrasts with other reports showing that lowering the LDL-C level through statin use significantly reduces the risk of major vascular events (risk ratio of 0.79 per 1.0 mmol/L reduction).^22^ The high prevalence of familial hypercholesterolaemia (FH) in Malaysia complicates achieving LDL-C control with statins alone.^23^ Moreover, the use of statins only is not associated with good LDL-C control. Patients with FH often need more aggressive treatments, including the addition of other LLDs. Apart from statins, achieving LDL-C treatment targets may require combination therapy with addon non-statins such as ezetimibe, bile acid sequestrants and proprotein convertase subtilisin/kexin type 9 inhibitors.^12^ Clinical trials, such as the IMPROVE-IT study, have shown that adding ezetimibe to statin therapy can further reduce the LDL-C level and CV event risk.^12^ However, government healthcare clinics in Malaysia currently offer limited LLDs, primarily statins and fibrates, which may not fully address the needs of patients with complex dyslipidaemia.^24^ This limitation underscores the need for incorporating alternative agents such as ezetimibe into treatment protocols to address the diverse needs of patients with dyslipidaemia, achieve LDL-C control and improve overall CV outcomes.

This study discovered that good BP control was associated with an age of >60 years and a DM duration of >10 years. This is likely due to more comprehensive management plans, frequent healthcare interactions and better self-management skills in patients.^15,25^

These factors enhance medication adherence and lifestyle changes, positively impacting BP control. Additionally, age-related vascular and autonomic changes may improve medication effectiveness. Sex differences in BP control reveal that men show poorer control than women. This disparity is attributed to poorer health-seeking behaviour and medication adherence in men as well as biological differences such as the protective effects of oestrogen in premenopausal women.^26^ Obesity contributes to poorer BP control through mechanisms including increased sympathetic nervous system activity, altered renal function and increased insulin resistance. Adipose tissue secretes substances that cause vascular dysfunction and elevated BP. Poorer BP control in patients on statins may reflect their higher CV risk and more complex health profiles. Statin use often indicates severe or resistant hypertension and multiple comorbidities, complicating BP management.^15^ Clinical focus on lipid management might overshadow aggressive BP control, leading to suboptimal outcomes. In contrast, patients not on statins are typically in early DM stages with less complicated treatment needs.

In the current study, good DM control was linked to an age of ≥60 years and age at diagnosis of ≥60 years. This may be due to better adherence to medical advice and more frequent healthcare interactions, leading to improved self-management skills. In contrast, poorer DM and LDL-C control was observed in the Malay and Indian patients than in the Chinese patients, likely due to differences in dietary habits, socioeconomic factors and healthcare access.^25^ This finding is supported by previous reports showing that Malays have the worst CV RF control.^27^ The relationship between ethnicity differences and CV RF control is more closely related to patients’ ethnicity than to any other factor.^28^ Dietary practice, socioeconomic level, religious and cultural beliefs, clinicians and health centre structures have a significant effect on DM control as part of CV RFs.^28,29^ Additionally, patients with a DM duration of ≥10 years struggle with glycaemic control, reflecting the progressive nature of the disease and the development of complications over time. Patients on two or three GLDs also show poorer control, suggesting that these individuals have more severe or resistant DM, requiring complex treatment regimens that are more difficult to manage effectively.^15^ This highlights the challenge of achieving optimal glycaemic control in patients with advanced or difficult-to-manage DM.

The main strength of this study is the revelation of SPM prescription and its association with RF control among patients with DM and CVDs. The findings revealed that despite good SPM prescription, RF control is still poor, which should alarm primary care providers. Despite this strength, there are some limitations to this study. First, the study sample was obtained from the NDR audit list. Hence, this study may not be representative of all patients with DM and CVDs. Second, the type and dosage of SPMs were not explored. This has significant implications for RF control, especially on the LDL-C level, as moderate-to-high-intensity statins play an important role in achieving the LDL-C control target.^22^ Finally, information on the patients’ socioeconomic status, dietary habits, cultural beliefs or treatment compliance, which could be useful for further analysis, was not captured in this study. As for clinical implications, the findings of this study provide useful insights for healthcare providers to ensure the optimisation of SPM prescription and achieve CV RF control. Further, the availability of LLDs other than statins should be considered in government healthcare clinics, as these drugs have been shown to improve LDL-C control.

## Conclusion

The prescription of SPMs in patients with CVDs remains good and has improved in the last 5 years. However, the achievement of lipid treatment targets remains poor and unchanged. The use of statins is not associated with lipid control. It is necessary to improve the accessibility and availability of alternative lipid-lowering agents to enhance overall RF control, especially lipid control, in patients with DM and CVDs.

## Data sharing statement

Raw data are available only upon request with permission from the Ministry of Health Malaysia.

**How does this paper make a difference in general practice?**

- Primary care providers must improve secondary prevention medication prescription and achieve target risk factor control among patients with diabetes mellitus and cardiovascular diseases.
- The study identified ethnic disparities, with Malay and Indian patients having poorer diabetes mellitus and lipid control than Chinese patients. This emphasises the importance of considering cultural and socioeconomic factors in healthcare delivery.
- Inadequate lipid control with statins indicates the need for optimised treatments, potentially with adjunct agents such as ezetimibe for better outcomes.
- This study provides important insights for healthcare practitioners and policymakers to enhance secondary prevention strategies, especially in primary care.
